# American marsupials chromosomes: Why study them?

**DOI:** 10.1590/S1415-47572009005000084

**Published:** 2009-12-01

**Authors:** Marta Svartman

**Affiliations:** Departamento de Biologia Geral, Instituto de Ciências Biológicas, Universidade Federal de Minas Gerais, Belo Horizonte, MGBrazil

**Keywords:** marsupials, cytogenetics, chromosome evolution

## Abstract

Marsupials, one of the three main groups of mammals, are only found in Australia and in the American continent. Studies performed in Australian marsupials have demonstrated the great potential provided by the group for the understanding of basic genetic mechanisms and chromosome evolution in mammals. Genetic studies in American marsupials are relatively scarce and cytogenetic data of most species are restricted to karyotype descriptions, usually without banding patterns. Nevertheless, the first marsupial genome sequenced was that of *Monodelphis domestica*, a South American species. The knowledge about mammalian genome evolution and function that resulted from studies on *M*. *domestica* is in sharp contrast with the lack of genetic data on most American marsupial species. Here, we present an overview of the chromosome studies performed in marsupials with emphasis on the South American species.

## Marsupials: Evolutionary History and Taxonomy

The class Mammalia is traditionally divided into three main groups: Prototheria, comprising only three species of extant monotremes, Metatheria, in which all marsupials are grouped, and Eutheria, that reunites all the other 18 mammalian orders.

Besides typical mammalian features, as homeothermy, hairs and mammary glands, marsupials have biological characteristics that allowed their separation as a distinct group. Among them, which include dental, osteological, cranial and brain features, those related to the reproductive system are the most important. These include a bifid reproductive tract in females, which have double vagina and uterus, and an incomplete placenta. The gestation time is relatively short and the newborns are in an almost embryonic stage. The marsupium or pouch, used to name the group, is not its most important feature and is absent in several species (Stonehouse, 1977; Nowak, 1991).

A recent estimate which included molecular data from almost all extant mammalian groups led Bininda-Emonds *et al.* (2007) to propose that the split of Monotremata from the common mammalian ancestor occurred at 166.2 million years ago (mya), that the lineage of the marsupials would have diverged at 147.7 mya and that the four recognized placental superorders would have originated around 100 mya. The diversification of the marsupials was dated at 82.5 mya.

Regarding their evolutionary history, the traditional hypothesis was that marsupials originated during the Cretaceous in North America. From there, they would have migrated to South America, Antarctica and Australia (Clemens, 1977). Marsupial fossils were also found in Europe, Asia and North Africa, where there are no extant representatives of the group, indicating a broader geographical distribution in the past (Nowak, 1991). The discovery of the metatherian fossil *Sinodelphys szalayi* in northeastern China in a site dated at 125 mya changed this picture (Luo *et al.*, 2003). Eutherian fossils have also been found in the same site, including *Eomaia*, the earliest known member of the eutherian lineage. Thus the marsupial-placental split must have been no later than 125 mya. *Sinodelphys* was found to be basal in the metatherian tree, which led Luo *et al.* (2003) to propose a Eurasian origin for both eutherians and metatherians. From Eurasia, marsupials would have migrated to North America and later to South America and then Antarctica and Australia.

The Australian groups of marsupials are phylogenetically and immunologically more closely related between them than they are to any American group. This led to the suggestion that they originated from a single ancestral stock that would have reached Australia in the beginning of the Tertiary (64 mya), coming from South America via Antarctica (Dawson, 1983). The phylogenetic relationship between the South American monito del monte *Dromiciops gliroides* and the Australasian marsupials is of special interest. The traditional view is that Australidelphia, a pan-Gondwanan clade that includes all extant Australian marsupials and the South American microbiotheres, represented presently only by *Dromiciops gliroides,* would have originated in South America.

Australian marsupials are believed to have reached that continent at the end of the Cretaceous (64 mya) and there they experienced an extensive radiation, which is suggested by the present diversity of the group in Australia. The oldest metatherian fossils known from Australia are from a single site dated at around 55 mya. The recent finding of fairly complete fossils from the metatherian *Djarthia murgonenis* led Beck *et al.* (2008) to propose that *Djarthia* was a member of Australidelphia. A phylogenetic analysis led to the conclusion that *Djartia* is the most plesiomorphic known australidelphian and may represent the ancestral morphotype of the Australian marsupial radiation. Beck *et al.* (2008) thus concluded that, contrary to the long held view, the South American microbiotheres may have resulted from back dispersal from marsupials from eastern Gondwana.

Marsupials have been traditionally grouped in the order Marsupialia, but the recognition of their great diversity is reflected in the increasing number of taxa proposed by different authors (Nowak, 1991; Wilson and Reeder, 2005). More recently new classifications were proposed mainly based on molecular data and the most current includes ten orders, seven of which with living representatives (Wilson and Reeder, 2005). These seven orders reunite 331 extant species, from which 237 inhabit Australasia and 94 occur in the American continent (Wilson and Reeder, 2005). The American marsupial fauna is predominantly found in Central and South America, as the only marsupial species in North America, *Didelphis virginiana*, is a relatively recent invader, derived from the South American *D*. *marsupialis* (Dawson, 1983).

Three marsupial orders each including one extant family are found in the American continent. Among them, the family Didelphidae, order Didelphimorphia, is the richest in number of taxa and has the broadest geographical distribution, with representatives in the three Americas. It has 87 species belonging to 17 genera (*Caluromys*, *Caluromysiops*, *Chironectes*, *Didelphis*, *Glironia*, *Gracilinanus*, *Hyladelphys*, *Lestodelphys,Lutreolina*, *Marmosa*, *Marmosops*, *Metachirus*, *Micoureus*, *Monodelphis*, *Philander*, *Thylamys* and *Tlacuatzin*). Six species, belonging to three genera (*Caenolestes*, *Lestoros* e *Rhyncolestes*), form the family Caenolestidae, order Paucituberculata, whose representatives inhabit high altitude regions, usually in the Andes; the family Microbiotheriidae, order Microbiotheria, has as its sole extant representative *Dromiciops gliroides*, which has only been found in southern Chile and in the adjacent region of Argentina (Wilson and Reeder, 2005).

## The Chromosomes of American Marsupials

Marsupials were one of the first mammalian groups to have their chromosomes studied. In his review, Hayman (1990) listed the diploid numbers of 178 species, which varies from 2n = 10 in *Pseudocheirus cupreus* to 2n = 32 in *Aepyprymnus rufescens*, both from Australia.

From the 94 currently recognized American marsupial species (Wilson and Reeder, 2005), 45 had their karyotypes reported, most of them without banding patterns (Table 1). Three diploid numbers have been found in American marsupials: 2n = 22 in species of *Didelphis*, *Chironectes*, *Philander* and *Lutreolina*, 2n = 18 in species of *Monodelphis*, and 2n = 14 in all other species studied, which belong to the genera *Caluromys*, *Gracilinanus*, *Marmosa*, *Metachirus*, *Thylamys*, *Micoureus*, *Marmosops*, *Tlacuatzin*, *Dromiciops*, *Caenolestes*, *Lestoros* and *Rhyncolestes*.

The current available chromosome data for American marsupials are detailed in Table 1. In this Table, the fundamental number (FN) seems to be variable both within the same species, for instance *Caluromys lanatus*, and among species with the same diploid number, as the *Monodelphis* species. A closer examination of the literature reveals that there is no real variation in autosomal arms in each of the species studied by each author. The reason for the difference is that some authors counted the short arms of the acrocentric or subtelocentric chromosomes, while others did not. When these short arms are not included, the FN count is very constant and equal to 20 in all species with 2n = 14, except for all the *Marmosops* species, the caenolestids and the microbiotherid, which exhibit a FN = 24. The difference in the FN between these two karyotypic forms is due to the two smaller autosome pairs, which are acrocentric in the FN = 20 and metacentric/submetacentric in the FN = 24 complements. The same explanation applies to the 2n = 18 *Monodelphis*: all the species studied presented two clearly biarmed elements, resulting in a FN = 20.

This observation means that no real variation was observed between the conventionally stained autosomes of specimens of the same species collected in different localities and studied by various authors. Moreover, no variation in the autosomes seems detectable among species with each of the three diploid numbers (2n = 14, 18 and 22), except for the species with 2n = 14 and FN = 24.

Great intraspecific conservation was also observed in the sex chromosomes. Many apparent differences are due to the nomenclature used by various authors (SM or M; T or A) or by technical difficulties because of the low quality of the chromosome preparations, especially in the older reports. The sex chromosomes are always the smallest ones in the karyotypes of the American marsupials studied and some species have very small Y chromosomes, considered as dot-like by some or as very small A by others. Due to their small size, determining their morphology may be difficult and variations in condensation complicates it even more.

The only cases of real intraspecific sex chromosome variation appear to be those of the X chromosomes of *Gracilinanus emiliae*, *G*. *microtarsus*, *Marmosamurina* and *Thylamys elegans*. Different morphologies were reported for these chromosomes by several authors (Table 1). Additionally, one specimen of *Philanderfrenatus* had a metacentric Y, in contrast with the acrocentric form found otherwise in this species (Carvalho *et al.*, 2002).

The Y chromosome was reported as undistinguishable or absent in males of *Thylamys pallidior* (Palma, 1995; Palma and Yates, 1996), *Marmosops noctivagus,Thylamys elegans* and *Chironectes minimus* (Palma and Yates, 1996). *T*. *pallidior* and *M*. *noctivagus* were only studied by these authors, but in *C*. *minimus* and *T*. *elegans* dot-like Y chromosomes were reported (Hayman and Martin, 1974; Reig *et al.*, 1972, 1977). It is thus very likely that the four species above have dot-like Y chromosomes that were missed for technical reasons by Palma (1995) and Palma and Yates (1996). Another such case is that of *Dromiciops gliroides*, which was reported to have no Y chromosome in male bone marrow cells, that would have a 2n = 13 (Gallardo and Patterson, 1987). Nevertheless, the Y chromosome of this species was also reported as dot-like in bone marrow cells in other studies (Reig *et al.*, 1977; Spotorno *et al.*, 1997) and was likely missed in the former report.

## Chromosome Banding In American Marsupials

G-banding was performed in 14 species of American marsupials (Sinha and Kakati, 1976; Yonenaga-Yassuda *et al.*, 1982; Merry *et al.*, 1983; Rofe and Hayman, 1985; Casartelli *et al.*, 1986; Souza *et al.*, 1990; Pathak *et al.*, 1993; Seluja *et al.*, 1984; Spotorno *et al.*, 1997; Svartman and Vianna-Morgante, 1999) (Table 1). No intraspecific variation was observed in these studies, which reinforced the conservation of the karyotypes extending it to G-banded chromosomes. Moreover, species with the same diploid number showed a striking similarity in their banded chromosomes with small karyotypic differences attributed to inversions and variation in the amount of constitutive heterochromatin (Yonenaga-Yassuda *et al.*, 1982; Rofe and Hayman, 1985; Casartelli *et al.*, 1986; Souza *et al.*, 1990; Svartman and Vianna-Morgante, 1999). The comparison of G-banded karyotypes of species with 2n = 14, 18 and 22 allowed to establish a complete homeology between all the autosomal arms of species with 2n = 14, 18 and 22 (Svartman and Vianna-Morgante, 1998, 1999). This observation reinforced the role played by Robertsonian rearrangements in the karyotypical evolution of American marsupials (Svartman and Vianna-Morgante, 1998, 1999).

C-banding patterns were obtained in 22 species of American marsupials (references in Table 1) and, although intraspecific variation seemed absent, differences in constitutive heterochromatin contents and location were reported among species with the same diploid numbers. Most of the ten 2n = 14 species studied presented pericentromeric heterochromatin in all autosomes and on the X chromosome (two species of *Caluromys*, two of *Gracilinanus*, *Marmosa murina* and *Thylamyselegans*). In *Metachirus nudicaudatus* only small pericentromeric C-bands were observed in the two smaller autosomes (pairs 5 and 6) and on the X chromosome. Large blocks of heterochromatin were present in the autosomes and on the X chromosomes of *Marmosops incanus* and *M*. *parvidens*. In *M*. *incanus* two additional large distal heterochromatic blocks were present, one in each arm of the metacentric X chromosome (Svartman and Vianna-Morgante, 1999; Carvalho *et al.*, 2002; Pagnozzi *et al.*, 2002). *Micoureus demerarae* had large heterochromatic blocks in the pericentromeric regions of the four largest autosome pairs (pairs 1-4) and very little pericentromeric C-bands in the two smallest autosomes (pairs 5 and 6). Besides the pericentromeric C-band, the acrocentric X chromosome of this species also presented a distal heterochromatic block (Casartelli *et al.*, 1986; Souza *et al.*, 1990; Svartman and Vianna-Morgante, 1999; Pagnozzi *et al.*, 2000; Carvalho *et al.*, 2002). The Y chromosome was described as entirely heterochromatic in all the species.

Four of the five *Monodelphis* species studied (*M*. *brevicaudata*, *M*. *dimidiata*, *M*. *domestica* and *M*. *kunsi*) presented pericentromeric heterochromatin in all autosomes and on the X chromosome and the tiny Y chromosome appeared completely heterochromatic. *Monodelphis americana* showed a different pattern, with pericentromeric heterochromatin restricted to the X chromosome and a completely heterochromatic Y (Merry *et al.*, 1983, Svartman and Vianna-Morgante, 1999; Carvalho *et al.*, 2002; Pagnozzi *et al.*, 2002).

In all species with 2n = 22 studied the Y chromosome appeared heterochromatic. The most common pattern observed was the presence of C-bands in the pericentromeric regions of all autosomes and on the X chromosome, which was the case in *Chironectes minimus*, *Didelphis albiventris*, *D*. *marsupialis* and *Philander frenatus*. Pericentromeric heterochromatin was restricted to the X chromosome in *Didelphis marsupialis*, *D*. *virginiana* and *Lutreolinacrassicaudata*. The two latter species presented distinctive features on their X chromosomes which had C-banded blocks. This was also the case for the X chromosome of *Philander opossum* (Sinha *et al.*, 1972; Sinha and Kakati, 1976; Yonenaga-Yassuda *et al.*, 1982; Seluja *et al.*, 1984; Casartelli *et al.*, 1986; Svartman and Vianna-Morgante, 1999; Carvalho *et al.*, 2002, Pagnozzi *et al.*, 2002).

The variation in X chromosome size among species including the three diploid numbers was attributed to variations in the contents of constitutive heterochromatin (Yonenaga-Yassuda *et al.*, 1982; Souza *et al.*, 1990; Svartman and Vianna-Morgante, 1999).

Genome comparisons among *Philander opossum* (2n = 22), *Micoureus demerarae* and *Marmosops incanus* (2n = 14) were extended through FISH (fluorescent *in situ* hybridization) using total genomic DNAs as probes (Svartman and Vianna-Morgante, 1999). In this study, interspecific conservation of the euchromatin among the three karyotypes was reinforced, while the heterochromatin was shown to be species-specific, not only in amount, but also in content.

Ag-NORs were studied in a total of 24 species of American marsupials. Thirteen of the species analyzed had a 2n = 14 (*Caluromys lanatus*, *C*. *philander*, *Gracilinanus emiliae*, *G*. *microtarsus*, *Marmosa murina*, *Marmosops incanus*, *M*. *parvidens*, *M*. *paulensis*, *Metachirus nudicaudatus*, *Micoureus demerarae*, *M*. *paraguayensis*, *Thylamys elegans* and *Dromiciops australis*; references in Table 1). All of these species presented Ag-NORs in the short arms of chromosome 6 (6p). In *Dromiciops gliroides* one additional NOR was observed in 5p (Fernandez-Donoso *et al.*, 1979) and in the two analyzed *Micoureus* species there were six NORs located on 5pq and 6p (Carvalho *et al.*, 2002; Svartman and Vianna-Morgante, 2003).

Five species of *Monodelphis*, all with 2n = 18, had their Ag-NORs described (*M*. *brevicaudata*, *M*. *dimidiata*, *M*. *domestica*, *M*. *kunsi* and *M*. *rubida*). Four of them presented a single NOR-bearing pair, which was 5p in three species (*M*. *dimidiata*, *M*. *kunsi* and *M*. *rubida*) and Xp in *M*. *brevicaudata*. *Monodelphisdomestica* was the only species with 2n = 18 to present four NORs, located on 5p and Xp (references in Table 1). It is possible that all *Monodelphis* species have the same pattern observed in *M*. *domestica* (5p and Xp) and that they were not detected because of the few specimens analyzed and because Ag-NORs reflect activity in the previous interphase and are thus variable. It would be interesting to perform FISH with an rDNA probe in order to determine the location of NORs in all the *Monodelphis* species. In *Monodelphisdomestica* there is no inactivation of the NOR on the X chromosomes of the females, which are both active in every cell analyzed (Merry *et al.*, 1983; Svartman and Vianna-Morgante, 2003). This may also be the case in *M*. *brevicaudata* (Carvalho *et al.*, 2002).

Three species with 2n = 22 presented four Ag-NORs located on 5p7q (*Lutreolina crassicaudata*, *Philander opossum* and *P*. *frenata*), while *Chironectes minimus* had a single Ag-NOR on 5p. In *Didelphis albiventris* the maximum number of Ag-NORs reported was six and they were located at the tip of the long arm of pairs 4, 5 and 6 (Seluja *et al.*, 1984). *Didelphismarsupialis* had eight NORs as shown by FISH with an rDNA probe (Svartman and Vianna-Morgante, 2003), but the number of Ag-NORs reported was highly variable, possibly reflecting differences in activity between samples (Yonenaga-Yassuda *et al.*, 1982; Casartelli *et al.*, 1986; Carvalho *et al.*, 2002; Svartman and Vianna-Morgante, 2003; Lima, 2004).

Besides *Monodelphis domestica and D*. *marsupialis*, other five species of American marsupials representing the three diploid numbers known in the group were studied after FISH with an rDNA probe (Svartman and Vianna-Morgante, 2003). The results obtained, combined with the demonstration of G-banding homeologies between all the autosomal arms of the three diploid numbers (2n = 14, 18 and 22), led to the conclusion that at least one NOR-bearing pair is the same in the three different 2n complements. Thus, the NOR-bearing chromosome 6 of the 2n = 14 karyotype corresponds, respectively, to chromosomes 5 and 7 in the species with 2n = 18 and 22, which also have NORs (Svartman and Vianna-Morgante, 2003).

## Karyotype Evolution In Marsupials

The preponderance of a conserved karyotype with 14 chromosomes in animals of almost all marsupial families from the American and Australian faunas was interpreted as evidence that this was a conserved complement, possibly present in a common ancestor. Martin and Hayman (1967) and Hayman and Martin (1969) suggested that a karyotype with 14 chromosomes would be the “ancestral karyotype” present in an ancestral stock. It was suggested that the evolution proceeded from a karyotype with 14 chromosomes, which would have undergone centric fissions originating the second mode, a complement with 22 chromosomes, which in turn, would have given rise to the karyotypes with intermediate diploid numbers through centric fusions (Hayman, 1990). A karyotypical evolution through centric fissions from the basic karyotype with 2n = 14 was also suggested to explain the 2n = 18 and 2n = 22 in American marsupials (Reig *et al.*, 1977).

An opposing view was held by Sharman (1973), who suggested that the second modal number of 2n = 22 would be ancestral as it was present in the more plesiomorphic American didelphids and because chromosome fissions are rarer and more difficult to accept. This author thus proposed that chromosome fusions would explain the occurrence of marsupial karyotypes with diploid numbers lower than 2n = 22.

The later demonstration that the G-banding patterns in American and Australian species with 2n = 14 were very similar, mainly differing due to intrachromosomal variations, was used to reinforce the idea that this was the marsupial “basic ancestral karyotype” and to lend support for the fission hypothesis (Rofe and Hayman, 1985; Hayman, 1990).

Recent studies using reciprocal interspecific chromosome painting to compare the chromosomes of marsupials from Australian and South American families confirmed the great conservation between chromosome segments among very divergent groups and allowed to delineate conserved segments that were differently combined in each species (Rens *et al.*, 1999; 2001; 2003). These studies, which confirmed and extended the conclusions based on previous G-banding comparisons (Rofe and Hayman, 1985; Yonenaga-Yassuda *et al.*, 1982; Souza *et al.*, 1990; Svartman and Vianna-Morgante, 1999) have been frequently used in support of the fission hypothesis. Nevertheless, the demonstration of conservation does not in itself favor any of the two competing views.

The finding of interstitial telomeric sequences (ITS) in the autosomes of Didelphidae species with 2n = 14 and 2n = 18, but not in those with 2n = 22, led to the suggestion that the karyotype of South American marsupials would have proceeded through centric fusions leading to the reduction in diploid number from an ancestral complement with at least 22 chromosomes (Svartman and Vianna-Morgante, 1998; Carvalho and Mattevi, 2000). This hypothesis defied the idea of the “ancestral” 2n = 14 marsupial karyotype which had prevailed for the previous three decades.

Pagnozzi *et al.* (2000) demonstrated that in *Micoureus demerarae* (2n = 14) autosomal pericentromeric interstitial telomeric sequences varied from 2 through 8 in specimens caught in different localities. These authors also stated that the ITS co-localized with heterochromatic blocks in *M*. *demerarae* and would thus represent part of satellite DNA sequences and not remnants of Robertsonian fusions. Pagnozzi *et al.* (2002) later performed the same kind of analysis in eight Didelphidae species, four with 2n = 14, two with 2n = 18 and two with 2n = 22, and came to a similar conclusion. These authors claimed that other kinds of datasets, basically derived from molecular studies involving DNA sequence comparisons, supported the ancestral 2n = 14 karyotype hypothesis and an evolution based on chromosome fissions would comply with it.

Taken together, the studies with ITS performed in American marsupials revealed conserved results in the six 2n = 22 species analyzed (*Chironectes minimus*, *Didelphis albiventris*, *D*. *marsupialis*, *Philanderfrenatus*, *P*. *opossum* and *Lutreolina crassicaudata*), that presented no ITS. In three out of the four species with 2n = 18 studied (*Monodelphis brevicaudata*, *M*. *domestica* and *M*. *kunsi*), ITS were conserved in the pericentromeric region of pair 1. The results in the eight 2n = 14 species analyzed were more variable: four species had no ITS (*Caluromys philander*, *Metachirus nudicaudatus*; *Marmosa murina* and *Gracilinanus emiliae*), *Gracilinanus microtarsus* had ITS on pair 1, *Micoureus demerarae* had a maximum of eight signals on the four largest autosome pairs, *Marmosopsincanus* had ITS on pairs 1-5 and *M*. *parvidens* presented ITS in all of its six autosomal pairs (Svartman and Vianna-Morgante, 1998; Carvalho and Mattevi, 2000; Pagnozzi *et al.*, 2000, 2002).

It is interesting to point out that ITS were only observed in biarmed chromosomes. Another point to be stressed is that, with the exception of *Marmosops incanus* and *Metachirus nudicaudatus* studied by Svartman and Vianna-Morgante (1998), the results obtained by the other authors were from metaphases derived from bone marrow cells. These spreads present relatively condensed chromosomes when compared with those derived from cultured fibroblasts. A closer look at the results obtained by Svartman and Vianna-Morgante (1998) clearly show that the ITS detected by FISH in *Marmosops incanus* are close to but not inside the pericentromeric heterochromatin, as suggested by Pagnozzi *et al.* (2002).

Although the fusion hypothesis was clearly advanced to account for the karyotypic evolution of the three diploid numbers in American marsupials (Svartman and Vianna-Morgante, 1998; Carvalho and Mattevi, 2000) Metcalfe *et al.* (2004) studied the distribution of ITS and of constitutive heterochromatin in nine species of Australian marsupials to test this view. Three of the species analyzed presented the presumed “ancestral” 2n = 14 karyotype and the other six species were Macropodinae with varying diploid numbers which had been previously extensively studied. Centromeric ITS were observed in the largest three chromosome pairs of the 2n = 14 species and also on pair 6 in two of them. In the Macropodinae species large ITS signals were observed in almost all chromosomes and the same sites were shown to be C-band positive. *Macropus agilis*, the only species without extensive C-banding, presented ITS in three pairs which corresponded to fusion sites from the putative 2n = 22 ancestral macropodine complement. The authors associated the presence of most ITS with heterochromatin, but attributed the ITS observed in *Macropus agilis* to be the result from previously postulated chromosome fusions. Based on these results, the authors concluded that ITS represent telomeric sequences that are part of the native satellite DNA and that they only represent remnants of rearrangements when not present in the heterochromatin, a conclusion similar to that advanced by Pagnozzi *et al.* (2000, 2002).

In conclusion, it seems that the support given by various authors to each of the alternative hypotheses (fission or fusion) is strongly influenced by their view on how the chromosome data should be interpreted. Thus, those who believe that chromosome data are accessory to other kinds of datasets and should be used to corroborate them favor the fission hypothesis. Contrarily, authors that tend to interpret chromosome data independently before comparison with other kinds of studies are more inclined to support the fusion hypothesis. In any case, the resolution of this debate will rely on more detailed studies aiming at a molecular characterization of the ITS in marsupials. The sequencing of these regions could reveal if their composition is strictly telomeric or if they are part of satellite DNAs located at the heterochromatin. Another possibility is to verify if the composition of the satellite DNAs present in the heterochromatin of different species is similar and contain telomere sequences embedded in them.

## Marsupials and Sex Chromosomes

Among the main contributions resulting from marsupial genetics studies are those related to sex chromosomes evolution and function. Sex determination in eutherians depends on the presence of the Y chromosome. Testis development is primarily due to the *SRY* gene and other male characters develop later under the control of the testicular hormones. Testes development in marsupials is also determined by the Y chromosome, which bears an orthologue of the human *SRY* gene (Foster *et al.*, 1992). Nevertheless, as demonstrated by Shaw *et al.* (1990), in marsupials the scrotum develops before the testes and thus, differently from eutherians, scrotum differentiation is not dependent on the hormones secreted by the developed testis.

Like most mammals, the majority of marsupials present a chromosome sex determination system of the XX:XY type. Multiple sex chromosomes systems were only described in four Australian species: in *Potoroustridactylus*, *Wallabia bicolor* and *Macrotis lagotis* the multiple sex chromosomes result from a translocation of an autosome to the X chromosome, whereas *Lagorchestesconspicillatus* presents a more complex system, involving translocations between two autosomes and both sex chromosomes (reviewed in Hayman, 1990).

Sex chromosome mosaicism resulting from their elimination in somatic tissues was described in several species. Almost all specimens from the Australian family Peramelidae (genus *Echymipera*, *Isoodon*, *Perameles* and *Peroryctes*) analyzed had one X chromosome eliminated in females and the Y chromosome eliminated in the same somatic tissues in males. The Y chromosome was absent in most cells of the bone marrow, liver and spleen of *Petauroides volans* (family Petauridae), but cells of the same tissue retained both X chromosomes in females (Murray and McKay, 1979).

A mechanism similar to that of *P*. *volans*, with the absence of the Y chromosome in bone marrow cells, was described in the American marsupial *Dromiciops gliroides* and used as evidence of its close phylogenetic relationship to Australian marsupials (Gallardo and Patterson, 1987). Nevertheless, the tiny Y chromosome of *D*. *gliroides* was probably missed by the authors, as discussed above in a previous topic.

Some cytological features of marsupial sex chromosomes are peculiar: the very small Y chromosome, which is dot-like in many species, and the small size of the X chromosome (3% of the genome) in relation to the eutherian X (5% of the genome) (Hayman *et al.*, 1982); the absence of the synaptonemal complex between the X and Y chromosomes during meiosis, which may be due to the absence of the pseudoautosomal region in marsupials (Sharp, 1982); the presence of nucleolus organizer regions (NORs) in the X or in both sex chromosomes of some species without their inactivation on one of the X chromosomes in females, which implies in escape from inactivation and no dosage compensation for these loci (Hayman, 1990; Svartman and Vianna-Morgante, 2003); and the differential location of chiasmata in the meiosis of males and females of some species (interstitial chiasmata in males and distal chiasmata in females, resulting in a much higher recombination rate in males), as *Sminthopsis crassicaudata* and *Monodelphis domestica* (Bennett *et al.*, 1986; Hayman *et al.*, 1988; Samollow *et al.*, 2004).

The absence of a synaptonemal complex between the X and Y chromosomes during the first meiotic prophase in males of the South American marsupials *Thylamys elegans,Dromiciops gliroides* and *Rhyncholestes raphanarus*, was demonstrated by Page *et al.* (2003, 2005). Instead, a structure called dense plate, a modification of the axial elements of the sex chromosomes, forms between the X and Y chromosomes during pachytene, thus ensuring their correct segregation.

The inactivation of the X chromosome in marsupials also presents important differences in relation to that of eutherians. In the somatic cells of eutherian females one X chromosome is randomly inactivated. This inactivation is stable through cell generations and encompasses most genes on the X chromosome. In marsupials and monotremes, there is preferential inactivation of the paternal X chromosome, which is incomplete and tissue specific (Cooper *et al.*, 1990; Heard and Disteche, 2006). The mechanisms involved in this inactivation are just starting to be understood (Hornecker *et al.*, 2007; Namekawa *et al.*, 2007).

Among the studies performed in marsupials, gene mapping was especially fruitful in providing important information related to chromosome evolution in mammals, particularly about the sex chromosomes.

Marsupials were very important in the identification of the mammalian testis determining factor (TDF) on the Y chromosome. The autosomal location of the putative TDF, the *ZFY* gene, in marsupials, was a strong indication that this was not the TDF gene (Sinclair *et al.*, 1988). The later finding of *SRY*, another candidate for TDF, on the marsupial Y chromosome, was used as evidence to corroborate its role as the mammalian TDF (Foster *et al.*, 1992).

Mapping of human X-linked genes in marsupials resulted in important findings. Genes already mapped in the human Xq were also mapped in the marsupial and monotreme X chromosomes, defining a mammalian X-conserved region (XCR). Nevertheless, human Xp genes were located in three autosomal regions in both marsupials and monotremes (reviewed in Wilcox *et al.*, 1996 and in Graves, 2006).

Based on the sex chromosomes mapping data and behavior in the three mammalian groups, Graves (1995) suggested that the eutherian sex chromosomes evolved through cycles of autosome additions to the ancestral X and Y chromosomes followed by the degradation of Y chromosome sequences and the recruitment of the equivalent regions on the X by the inactivation mechanism. The pseudoautosomal region on the human sex chromosomes would be a relict of the last addition and the genes on the Y chromosome without an essential function in sex determination and differentiation in males would tend to mutate or to be deleted (Graves, 1995; reviewed in Graves, 2006).

Gene mapping results in marsupials contradicted the hypothesis of mammalian X chromosome conservation proposed by Ohno (1967), as only part of the X was conserved in mammals. The same results also weakened the idea proposed by Lyon (1974) that the loss of Y chromosome genes and X chromosome inactivation could have happened in a single deletion or translocation event, as suggested by the lack of variation in size and gene content of the mammalian X chromosome.

Gene mapping data in American marsupials were much more limited than those from Australian species. Until a decade ago, *Didelphis virginiana* was the only American marsupial species to have genes mapped by *insitu* hybridization. In this case, *G6PD* was located on the Xp and *HPRT* was mapped on the Xq (Driscoll and Migeon, 1988). These genes are in the region considered to be conserved on the X chromosome of all species of eutherians, marsupials and monotremes already studied. They are closely located in eutherians and more distantly located in marsupials. In most species of Dasyuridae and Didelphidae the X chromosome is a small acrocentric, considered to be the marsupial ancestral X (Rofe and Hayman, 1985). The X chromosome of *D*. *virginiana* is submetacentric and probably originated from a pericentric inversion in the *D*. *marsupialis* X chromosome, which would explain the location of *G6PD* and *HPRT* in different X chromosome arms (Driscoll and Migeon, 1988).

Recently, the lack of more detailed data from American marsupials changed radically with the publication of the genome sequence of *Monodelphisdomestica*, the first marsupial species to be sequenced (Mikkelsen *et al.*, 2007). This species has been used as a model laboratory animal for biomedical research for some decades and, although originally from South America, has been studied by scientists worldwide. The contributions provided by the study of *M.domestica* for different biological areas along the years were recently reviewed by Samollow (2008) and include very important data on mammalian genetics.

In conclusion, studies already performed in marsupials, particularly in Australian species, have demonstrated the great potential provided by the group for the understanding of basic genetic mechanisms in mammals. Genetic studies in American marsupials are relatively scarce and cytogenetic data of most species are restricted to karyotype description, usually without banding patterns. We hope to see this situation change by having South American researchers interested in studying marsupials, which are not only an important component of our biodiversity, but a treasure trove of information on genome function and evolution.

## Figures and Tables

**Table 1:** American marsupial species karyotyped.

Species^a^	Referred as	2n	FN	X	Y	Banding and FISH	Provenience^b^	Reference
Family Didelphidae, Order Didelphimorphia								
*Caluromys derbianus*		14	24	A	T		Nicaragua	Biggers *et al.*, 1965
		14	24	A	A		Cartago, Costa Rica; Chiapas, Mexico	Reig *et al.*, 1977

*Caluromys lanatus*		14	24	SM	-		Iquitos, Peru	Hayman and Martin, 1974
		14	24	A	A		Loreto, Peru	Reig *et al.*, 1977
		14	22	M	-	G, C, Ag-NORs	San Diego, Zoo, USA	Rofe and Hayman, 1985
		14	22	SM	A	G, Ag-NORs	Amazonas, Brazil	Casartelli *et al.*, 1986
		14	20	A	D	G, Ag-NORs	Rondônia, Brazil	Souza *et al.*, 1990
		14	22	A	M		Pando, Bolivia	Palma and Yates, 1996
		14	20	SM	D	Ag-NORs	Goiás, Brazil	Pereira *et al.*, 2008

*Caluromys philander*		14	24	A	-		not specified	Hayman and Martin, 1974
		14	24	SM	A		Simla, Trindad; Guárico, Aragua and Miranda, Venezuela	Reig *et al.*, 1977
		14	20	A	D	G, C, Ag-NORs	Pernambuco, Brazil	Souza *et al.*, 1990
		14	20	A	A	G, Ag-NORs, FISH (Tel; rDNA)	São Paulo, Brazil	Svartman and Vianna-Morgante, 1998, 1999, 2003
		14	20	A	D	Ag-NORs	São Paulo, Brazil	Pereira *et al.*, 2008

*Gracilinanus agilis*		14	24	SM	SM		La Paz, Bolivia	Palma and Yates, 1996
		14	24	M	A		Goiás and Minas Gerais, Brazil	Carvalho *et al.*, 2002

*Gracilinanus emiliae*		14	24	M/A*	A	C, Ag-NORs, FISH (Tel)	Goiás, Brazil	Carvalho and Mattevi, 2000; Carvalho *et al.*, 2002
		14	24	SM	A	Ag-NORs	Goiás, Brazil	Pereira *et al.*, 2008

*Gracilinanus microtarsus*		14	24	SM	A	C, Ag-NORs, FISH (Tel)	Rio Grande do Sul, Brazil	Carvalho and Mattevi, 2000; Carvalho *et al.*, 2002
		14	20	A	A		Bahia, Brazil	Pereira and Geise, 2007
		14	24	M	-		São Paulo, Brazil	Pereira *et al.*, 2008

*Marmosa mexicana*		14	24	A	D		Nicaragua	Biggers *et al.*, 1965

*Marmosa murina*		14	24	M	A		Villavicencio, Colombia	Hayman and Martin, 1974
		14	24	M	A		Loreto, Peru; Bolivar, Venezuela	Reig *et al.*, 1977
		14	20	A	D	G, C, Ag-NORs	Pernambuco, Brazil	Souza *et al.*, 1990
		14	24	M/A*	A	C, Ag-NORs, FISH (Tel)	Amapá, Goiás and Tocantins, Brazil	Carvalho and Mattevi, 2000; Carvalho *et al.*, 2002
		14	20	A	-	C, FISH (Tel)	Ceará and Mato Grosso, Brazil	Pagnozzi *et al.*, 2002
		14	22	A	-	Ag-NORs	Tocantins, Brazil	Lima, 2004
		14	20	A	D		Espírito Santo, Brazil	Paresque *et al.*, 2004
		14	20	SM	A	Ag-NORs	Goiás and Tocantins, Brazil	Pereira *et al.*, 2008

*Marmosa robinsoni*		14	20	M	T		Guárico and Apure, Venezuela	Reig, 1968
		14	24	SM	A		Guárico, Apure and Miranda, Venezuela	Reig *et al.*, 1977

*Marmosops dorothea*		14	24	M	A		La Paz, Bolivia	Palma and Yates, 1996

*Marmosops fuscatus*	*Marmosa fuscata*	14	24	M	A		Northern Venezuela	Reig and Sonneschein, 1970
	*Marmosa fuscata*	14	24	M	A		Aragua and Miranda, Venezuela	Reig *et al.*, 1977

*Marmosops incanus*		14	24	M	A	G, C, Ag-NORs, FISH (Tel; rDNA; Gen)	São Paulo, Brazil	Svartman and Vianna-Morgante, 1998, 1999, 2003
		14	24	M	A	C, Ag-NORs	Bahia and Minas Gerais, Brazil	Carvalho *et al.*, 2002
		14	24	M	-	C, FISH (Tel)	Bahia and São Paulo, Brazil	Pagnozzi *et al.*, 2002
		14	24	M	A		Espírito Santo, Brazil	Paresque *et al.*, 2004
		14	24	M	A		Bahia, Brazil	Pereira and Geise, 2007
*Marmosops noctivagus*		14	24	SM	UN		La Paz, Bolivia	Palma and Yates, 1996
*Marmosops parvidens*		14	24	SM	-		La Paz, Bolivia	Palma and Yates, 1996
		14	24	SM	A	Ag-NORs	Goiás, Brazil	Carvalho *et al.*, 2002
		14	24	M	A	C, FISH (Tel)	Mato Grosso, Brazil	Pagnozzi *et al.*, 2002

*Marmosops paulensis*		14	24	M	A	Ag-NORs	São Paulo, Brazil	Pereira *et al.*, 2008

*Metachirus nudicaudatus*		14	20	A	-		Kasmera, Venezuela	Hayman and Martin, 1974
		14	20	A	A	C	Meta, Colombia	Yunis *et al.*, 1973
		14	24	A	A		Loreto and Ayacucho, Peru;Trujillo and Mérida, Venezuela	Reig *et al.*, 1977
		14	20	A	-		La Paz, Bolivia	Palma and Yates, 1996
		14	20	A	A	G, C, Ag-NORs, FISH (Tel; rDNA)	São Paulo, Brazil	Svartman and Vianna-Morgante, 1998, 1999, 2003
		14	20	A	A	C, FISH (Tel)	Mato Grosso, Brazil	Pagnozzi *et al.*, 2002
		14	20	A	A		Espírito Santo, Brazil	Paresque *et al.*, 2004
		14	20	A	-	Ag-NORs	São Paulo, Brazil	Pereira *et al.*, 2008

*Micoureus alstoni*	*Marmosa alstoni*	14						Hsu and Bernischke, 1971^c^
	*Marmosa alstoni*	14	24	A	A		San Jose, Costa Rica	Reig *et al.*, 1977

*Micoureus constantiae*		14	20	A	A		Pando, Bolivia	Palma and Yates, 1996

*Micoureus demerarae*		14	20	A	-		Rancho Grande, Venezuela	Hayman and Martin, 1974
	*Marmosa cinerea*	14	24	M	A		Loreto, Peru; Aragua, Bolivar, Amazonas and Mérida, Venezuela	Reig *et al.*, 1977
	*Marmosa cinerea*	14	20	A	A	G, C, Ag-NORs	Amazonas, Brazil	Casartelli *et al.*, 1986
	*Marmosa cinerea*	14	20	A	A	G, C, Ag-NORs	Pernambuco, Brazil	Souza *et al.*, 1990
	*Micoureus cinereus*	14	20	A	-		La Paz, Bolivia	Palma and Yates, 1996
		14	20	A	A	G, C, Ag-NORs, FISH (Tel; rDNA, Gen)	São Paulo, Brazil	Svartman and Vianna-Morgante, 1998, 1999, 2003
		14	20	A	A	C, FISH (Tel)	Bahia, Ceará, Goiás, Mato Grosso and São Paulo, Brazil	Pagnozzi *et al.*, 2000
		24	24	A	A	C, Ag-NORs, FISH (Tel)	Goiás and Rio Grande do Sul, Brazil	Carvalho and Mattevi, 2000; Carvalho *et al.*, 2002
		14	20	A	A		Espírito Santo, Brazil	Paresque *et al.*, 2004
		14	20	A	-	Ag-NORs	São Paulo, Brazil	Pereira *et al.*, 2008

*Micoureus paraguayanus*		14	20	A	-	Ag-NORs	São Paulo, Brazil	Pereira *et al.*, 2008
*Thylamys elegans*	*Marmosa elegans*	14	24	SM	D		Aconcagua, Chile	Reig *et al.*, 1972
	*Marmosa elegans*	14	22	SM	A		Aconcagua, Chile; Huancavelica and Arequipa, Peru	Reig *et al.*, 1977
	*Marmosa elegans*	14	24	A	-	Ag-NORs	Coquimbo, Chile	Fernandez-Donoso *et al.*, 1979
		14	20	A	UN		Cochabamba and Santa Cruz, Bolivia	Palma and Yates, 1996
		14	20	ST	-	G, C	Rio Loa, Pichidangui and Las Melosas, Chile	Spotorno *et al.*, 1997

*Thylamys macrurus*	*T. macrura*	14	20	A	-		Concepción, Paraguay	Palma, 1995

*Thylamys pallidior*		14	20	A	UN		Chuquisaca and Tarija, Bolivia	Palma, 1995
		14	20	A	UN		Chuquisaca, Bolivia	Palma and Yates, 1996

*Thylamys pusillus*	*Marmosa pusilla*	14	24	M	D		Buenos Aires, Argentina	Reig *et al.*, 1977
		14	20	SM	-		Tarija, Bolivia	Palma and Yates, 1996

*Thylamys velutinus*		14	24	SM	A		Goiás, Brazil	Carvalho *et al.*, 2002

*Monodelphis americana*		18	22	T	-		Paraíba, Brazil	Langguth and Lima, 1988
		18	22	A	A	C, FISH (Tel)	Ceará, Brazil	Pagnozzi *et al.*, 2002
		18	32	A	A		Espírito Santo, Brazil	Paresque *et al.*, 2004
		18	32	A	-		São Paulo, Brazil	Pereira *et al.*, 2008

*Monodelphis brevicaudata*		18	20	A	D		Aragua and Zulia, Venezuela	Reig and Bianchi, 1969
		18	30	A	A		Aragua, Merida and Zulia, Venezuela	Reig *et al.*, 1977
	*M. orinoci*	18	30	A	A		Guárico, Venezuela	Reig *et al.*, 1977
		18	22	A	D		Pando, Bolivia	Palma and Yates, 1996
		18	30	A	D	C, Ag-NORs, FISH (Tel)	Amapá and Roraima, Brazil	Carvalho and Mattevi, 2000; Carvalho *et al.*, 2002

*Monodelphis dimidiata*		18	20	A	D		Buenos Aires, Argentina	Reig and Bianchi, 1969
		18	30	A	A		Buenos Aires, Argentina	Reig *et al.*, 1977
		18	32	SM	D	C, Ag-NORs	Rio Grande do Sul, Brazil	Carvalho *et al.*, 2002

*Monodelphis domestica*		18	20	A	A	G, C, NOR		Merry *et al.*, 1983
		18	20	A	A	G	Laboratory bred, USA	Pathak *et al.*, 1993
		18	24	A	A		Chuquisaca and Santa Cruz, Bolivia	Palma and Yates, 1996
		18	20	A	-	G, C, Ag-NORs, FISH (Tel; rDNA)	Laboratory bred, Brazil	Svartman and Vianna-Morgante, 1998, 1999, 2003
		18	28	A	D	C, Ag-NORs, FISH (Tel)	Goiás, Brazil	Carvalho and Mattevi, 2000; Carvalho *et al.*, 2002
		18	20	A	A	C, FISH (Tel)	Ceará and Goiás, Brazil	Pagnozzi *et al.*, 2002
		18	30	A	D		Espírito Santo, Brazil	Paresque *et al.*, 2004
		18	22	A	A		Bahia, Brazil	Pereira and Geise, 2007
		18	20	A	A		Goiás and Tocantins, Brazil	Pereira *et al.*, 2008

*Monodelphis kunsi*		18	30	SM	SM		Tarija, Bolivia	Palma and Yates, 1996
		18	30	SM	A	C, Ag-NORs, FISH (Tel)	Goiás, Brazil	Carvalho and Mattevi, 2000; Carvalho *et al.*, 2002

*Monodelphis rubida*		18	32	A	A	Ag-NORs	São Paulo, Brazil	Pereira *et al.*, 2008

*Chironectes minimus*		22	20	A	A		Rio Camoto, Venezuela	Hayman and Martin, 1974
		22	20	A	A		not specified	Reig *et al.*, 1977
		22	20	A	UN		La Paz, Bolivia	Palma and Yates, 1996
		22	20	A	-	C, Ag-NORs, FISH (Tel)	Goiás, Brazil	Carvalho and Mattevi, 2000; Carvalho *et al.*, 2002

*Didelphis albiventris*		22						Saez, 1931^c^
		22	20	A	A		Buenos Aires, Argentina; Mérida, Venezuela; Huanaco, Peru	Reig *et al.*, 1977
		22	20	A	A	G, C	São Paulo, Brazil	Yonenaga-Yassuda *et al.*, 1982
		22	20	A	A	G, C, Ag-NORs	Uruguay (six localities)	Seluja et. al., 1984
		22	20	A	A	G, C, Ag-NORs	São Paulo, Brazil	Casartelli *et al.*, 1986
		22	20	A	A		Tarija, Bolivia	Palma and Yates, 1996
		22	20	A	A	C, Ag-NORs, FISH (Tel)	Goiás and Tocantins, Brazil	Carvalho and Mattevi, 2000; Carvalho *et al.*, 2002

*Didelphis aurita*		22	20	A	A		Bahia, Brazil	Carvalho *et al.*, 2002
		22	20	A	A		Espírito Santo, Brazil	Paresque *et al.*, 2004

*Didelphis marsupialis*		22						Dreyfus and Campos, 1941^c^
		22	20	SM	T		Philadelphia, USA	Biggers *et al.*, 1965
		22	20	A	A		San Jose, Costa Rica; San Luis Potosi, Veracruz and Chiapas, Mexico; Loreto, Peru; Aragua, Miranda and Merida, Venezuela	Reig *et al.*, 1977
		22	20	A	A	G, C, Ag-NORs	São Paulo, Brazil	Yonenaga-Yassuda *et al.*, 1982
		22	20	A	A	G, Ag-NORs	Amazonas and São Paulo, Brazil	Casartelli *et al.*, 1986
		22	20	A	A		La Paz, Bolivia	Palma and Yates, 1996
		22	20	A	A	G, C, Ag-NORs, FISH (Tel; rDNA)	São Paulo, Brazil	Svartman and Vianna-Morgante, 1998, 1999, 2003
		22	20	A	A	C, Ag-NORs	Amapá and Pará	Carvalho *et al.*, 2002
		22	20	A	A	C, FISH (Tel)	Mato Grosso, Brazil	Pagnozzi *et al.*, 2002
		22	20	A	A	Ag-NORs	Tocantins, Brazil	Lima, 2004
		22	20	A	-		Tocantins, Brazil	Pereira *et al.*, 2008

*Didelphisvirginiana*		22						Painter, 1922^c^
		22	32	M	A	C	Texas, USA	Sinha *et al.*, 1972
		22	32	M	A	G, C, H^3^-timidine replication	not specified	Sinha and Kakati, 1976
		22	32	M	A		San Luis Potosi, Nayarit, Chiapas and Yucatán, Mexico; Louisiana and Texas, USA	Reig *et al.*, 1977

*Lutreolina crassicaudata*		22						Saez, 1938^c^
		22	20	M	A		Buenos Aires, Argentina	Reig *et al.*, 1977
		22	20	M	A	G, C	São Paulo, Brazil	Yonenaga-Yassuda *et al.*, 1982
		22	20	M	A	G, C, Ag-NORs	Uruguay (six localities)	Seluja et. al., 1984
		22	20	M	A		not spec, Bolivia	Palma and Yates, 1996
		22	20	M	A	C, Ag-NORs, FISH (Tel)	Rio Grande do Sul, Brazil	Carvalho and Mattevi, 2000; Carvalho *et al.*, 2002
		22	20	M	-	C, FISH (Tel)	Goiás, Brazil	Pagnozzi *et al.*, 2002

*Philander frenatus*	*P. frenata*	22	20	A	A/M	C, Ag-NORs, FISH (Tel)	Santa Catarina and Rio Grande do Sul, Brazil	Carvalho and Mattevi, 2000; Carvalho *et al.*, 2002
	*P. frenata*	22	20	A	A		Espírito Santo, Brazil	Paresque *et al.*, 2004
	*P. frenata*	22	20	A	A	Ag-NORs	São Paulo, Brazil	Pereira *et al.*, 2008

*Philander mcilhennyi*		22	20	A	A		Loreto, Peru	Reig *et al.*, 1977

*Philander opossum*		22	20	T	T		Nicaragua	Biggers *et al.*, 1965
		22	20	A	A		Cartago, Costa Rica; Chiapas, Mexico; Loreto and Ayacucho, Peru; Barinas, Venezuela	Reig *et al.*, 1977
		22	20	A	A	G, C, Ag-NORs	São Paulo and Rio de Janeiro, Brazil	Yonenaga-Yassuda *et al.*, 1982
		22	20	A	A		Beni, Chuquisaca and Santa Cruz, Bolivia	Palma and Yates, 1996
		22	20	A	-	G, C, Ag-NORs, FISH (Tel; rDNA; Gen)	Espírito Santo and São Paulo, Brazil	Svartman and Vianna-Morgante, 1998, 1999, 2003
		22	20	A	A	C, Ag-NORs, FISH (Tel)	Goiás, Brazil	Carvalho and Mattevi, 2000; Carvalho *et al.*, 2002
		22	20	A	A	Ag-NORs	Goiás, Brazil	Pereira *et al.*, 2008

*Tlacuatzin canescens*	*Marmosa canescens*	22	20	A	D		Mexico (four localities)	Engstrom and Gardner, 1988

Family Caenolestidae, Order Paucituberculata								
*Caenolestes fuliginosus*		14	24	A	D		not specified	Hayman *et al.*, 1971^d^
	*C. obscurus*	14	24	A	D		Cauca, Colombia	Hayman *et al.*, 1971

*Lestoros inca*		14	24	A	D		Cuzco, Peru	Hayman *et al.*, 1971

*Rhyncholestes raphanarus*		14	24	A	A		Osorno, Chile	Gallardo and Patterson, 1987

Family Microbiotheriidae, Order Microbiotheria								
*Dromiciops gliroides*	*D. australis*	14	24	A	-		Valdivia, Chile	Reig *et al.*, 1972
	*D. australis*	14	24	A	A		Valdivia, Chile	Reig *et al.*, 1977
	*D. australis*	14	24	A	-	Ag-NOR	Valdivia, Chile	Fernandez-Donoso *et al.*, 1979
	*D. australis*	14	24	A	UN		Valdivia, Osorno and Concepción, Chile	Gallardo and Patterson, 1987
		14	24	ST	D	G	Valdivia and Osorno, Chile	Spotorno *et al.*, 1997

2n - diploid number; FN - fundamental number (number of autosomal arms); D - dot-like; A- acrocentric; T - telocentric; SM - submetacentric; M - metacentric; FISH - fluorescent *in situ* hybridization: Tel - telomere probe; rDNA - ribosomal DNA probe, Gen - whole genomic DNA probe; * - polymorphism of the X chromosome; UN - undistinguishable; a - current taxonomic names according to Wilson and Reeder, 2005; b - the provenience corresponds to states, departments or provinces in each country indicated; c - *apud* Hayman, 1990; d - in the original paper two *Caenolestes* species (*C*. *obscurus* and *C*. *fulliginosus*) were described with identical karyotypes. They were likely the same species as *C*. *obscurus* is now considered a synonym of *C*. *fulliginosus*.
